# The association between glycaemic control during hospitalization and risk of adverse events: A retrospective cohort study

**DOI:** 10.1002/edm2.268

**Published:** 2021-05-27

**Authors:** Lan Deng, Wusiman Aibibula, Zahra Talat, Kristian B. Filion, Shaun Eintracht, Kaberi Dasgupta, Vicky Tagalakis, Agnieszka Majdan, Oriana Hoi Yun Yu

**Affiliations:** ^1^ Department of Medicine McGill University Montreal QC Canada; ^2^ Center for Clinical Epidemiology Lady Davis Institute Jewish General Hospital Montreal QC Canada; ^3^ Department of Epidemiology, Biostatistics and Occupational Health McGill University Montreal QC Canada; ^4^ Department of Internal Medicine Division of Medical Biochemistry Jewish General Hospital McGill University Montreal QC Canada; ^5^ Divisions of Internal Medicine, Endocrinology and Metabolism, and Epidemiology Department of Medicine McGill University Health Centre Montréal QC Canada; ^6^ Centre for Events Research and Evaluation (CORE) Research Institute of the McGill University Health Centre Montréal QC Canada; ^7^ Department of Internal Medicine Jewish General Hospital McGill University Montreal QC Canada; ^8^ Division of Endocrinology Jewish General Hospital Montreal QC Canada

**Keywords:** adverse events, cohort study, glycaemic target

## Abstract

**Introduction:**

Hyperglycaemia is common during hospitalization; glycaemic targets in non‐critical care settings have not been well studied. We assessed associations between inpatient glycaemic control and adverse events.

**Methods:**

We conducted a retrospective cohort study on non‐critically ill medical patients hospitalized in a tertiary care hospital between 2015 and 2018. Mean glycaemia during the first four days of hospitalization was categorized as 4.0–7.0 mmol/L, 7.1–10.0 mmol/L and >10.0 mmol/L. The primary outcome was a composite of adverse events including mortality, infections, acute kidney injury, thromboembolic and cardiovascular events. The secondary outcome was hypoglycaemia, defined as any glycaemia <4.0 mmol/L. Logistic regression was used to assess adverse events, and a Cox proportional hazards model was used to estimate hypoglycaemia risk.

**Results:**

Our cohort included 1,368 patients, of whom 407 (29.8%) experienced an adverse event. We did not find associations between glycaemia of 4.0–7.0 mmol/L (adjusted odds ratio [OR]: 0.88, 95% confidence interval [CI]: 0.63–1.23) or glycaemia of >10.0 mmol/L (adjusted OR: 0.98, 95% CI: 0.75–1.28) and the occurrence of adverse events, compared to a glycaemia of 7.1–10.0 mmol/L. Glycaemia of >10.0 mmol/L was associated with an increased risk of hypoglycaemia (adjusted hazard ratio [HR]: 1.72, 95% CI: 1.21–2.45). Hypoglycaemia was associated with adverse events (adjusted OR 1.85, 95% CI 1.31–2.60).

**Conclusions:**

Neither glycaemia of 4.0–7.0 mmol/L nor glycaemia of >10.0mmol/L during non‐critical care hospitalization was associated with increased adverse events. Glycaemia of >10.0 mmol/L was associated with increased hypoglycaemia, likely due to aggressive glucose lowering. These findings highlight the need for further studies to discern optimal inpatient glycaemic targets.

## Graphical abstract

Few studies have assessed the association between various glycaemic targets and adverse outcomes during hospitalization. Our study assessed whether glycaemia levels (ie 4.0–7.0 mmol/L, 7.1–10.0 mmol/L and >10 mmol/L) during early hospitalization is associated with an increased risk of adverse events and hypoglycaemia. Our results showed that the glycaemic targets defined in our study were not associated with an increased risk of adverse events during hospitalization; however, hypoglycaemia (ie <4.0 mmol/L) was associated with an increased risk of adverse events during hospitalization.

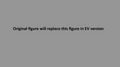

## 1 INTRODUCTION

Hyperglycaemia is common among hospitalized patients with a prevalence of up to 38%.^1^ Common causes for hyperglycaemia among hospitalized patients include increased secretion of stress hormones, use of glucocorticoids and failure to re‐initiate anti‐diabetic medications.^2^, ^3^ Studies have shown that hyperglycaemia in various clinical settings is associated with adverse patient outcomes, including infections, cerebrovascular and cardiovascular events, prolonged hospital stay and death.^1^, ^4^, ^5^, ^6^, ^7^, ^8^, ^9^, ^10^, ^11^ Potential mechanisms by which hyperglycaemia may lead to these adverse events include impairment of neutrophil and macrophage function, decreasing lymphocytes, enhancing platelet activation, decreasing tissue plasminogen activator and plasma fibrinolytic activity, and impairment of myocardial glucose utilization.^12^, ^13^, ^14^, ^15^, ^16^, ^17^ Furthermore, hyperglycaemia has been found to cause endothelial dysfunction and increase oxidative stress.^18^, ^19^ Some of these processes have been shown to improve with lowering of glucose levels to normal range.^20^, ^21^, ^22^

Nevertheless, in‐hospital glycaemic control is often neglected as care is focussed on the underlying presentation of illness.^23^ The American Diabetes Association recommends a target random blood glucose of 7.8 mmol/L to 10.0 mmol/L for the majority of hospitalized patients. More stringent goals between 6.1 and 7.8 mmol/L may be appropriate for selected patients if they can be achieved without significant hypoglycaemia. These targets are extrapolated from randomized controlled trials conducted mainly in the critically ill patient population.^24^ For non‐critically ill patients, the association between glycaemic control and adverse outcomes has not been extensively studied, and the limited number of studies conducted to date has inconsistent results.^25^ A meta‐analysis of 19 studies by Murad et al.^25^ reported no association between intensive glycaemic control and the risk of mortality, myocardial infarction or stroke among non‐critically ill hospitalized patients with diabetes; however, the studies included were heterogeneous and the evidence was mainly derived from surgical patients. To further understand the effects of glycaemic control during non‐critical care hospitalization in medical patients, we conducted a retrospective study to determine the association between glycaemic control and adverse events among medical patients admitted to non‐critical care units.

## 2 METHODS

### 2.1 Study design and population

We conducted a retrospective cohort study using electronic health records of patients treated at the Jewish General Hospital, a tertiary care teaching hospital for adult patients located in Montréal, Quebec, Canada. For each patient admitted to internal medicine units between 1 January 2015 and 31 December 2018, we obtained the discharge abstract from the medical records department and laboratory data from the biochemistry database. The discharge abstracts included the primary and secondary diagnoses of each patient, as well as new diagnoses and complications that arose during hospitalization documented by the treating physician. Patients carried a variety of common internal medicine admission diagnoses, including haematology‐oncology patients who required hospitalization. All medical diagnoses were recorded using International Classification of Diseases (ICD)‐10 codes. The study protocol was approved by the Research Ethics Committee at the Jewish General Hospital, Montréal, Canada.

We included patients aged 18 years or older with at least two capillary glucose measurements performed daily during the first four days of hospitalization. Capillary glucose measurements are performed routinely in patients with a history of diabetes during hospitalization, before meals and at bedtime, and more frequently should hypoglycaemia occur. As such, patients with various types of diabetes were included. Patients with diagnoses of pregnancy, diabetic ketoacidosis and non‐ketotic hyperglycaemic‐hyperosmolar state at the time of admission were excluded.

### 2.2 Exposure

The mean glycaemia during the first four days of hospitalization were calculated and classified into three categories for the purpose of this study: 4.0–7.0 mmol/L, 7.1–10.0 mmol/L (reference group) and >10.0 mmol/L. In an attempt to minimize protopathic bias, the first four days of glycaemic data were arbitrarily collected, based on previous studies that demonstrated length of medical hospitalization to be around 6 to 8.5 days.^8^, ^26^ Hypoglycaemia was defined as having any glycaemia less than 4.0 mmol/L during hospitalization.^24^, ^27^

During the study period, clinical practice in the management of hyperglycaemia was relatively unchanged. The only novel anti‐diabetic agent introduced was the sodium‐glucose cotransporter (SGLT)‐2 inhibitors which were available from 2 February 2015 in the Quebec public formulary.^28^, ^29^ At the Jewish General Hospital, there are standardized insulin sliding scale protocols that physicians generally prescribe for patients with diabetes. The insulin sliding scale protocol can be adjusted by the treating physician if necessary, to help prevent hypo‐ or hyperglycaemia during hospitalization.

### 2.3 Outcomes

The primary outcome was a composite of infections (urinary tract infection, pneumonia, Clostridium difficile and other infectious colitis, cellulitis, wound ulcer and sepsis), thromboembolic events (pulmonary embolism, deep vein thrombosis), cardiovascular events (myocardial infarction, unstable angina, ischaemic stroke and transient ischaemic attack), acute kidney injury and all‐cause mortality that occurred during hospitalization. The secondary outcome was hypoglycaemia, defined as having any glycaemia of <4.0 mmol/L anytime during the entire hospitalization, with the event date defined by the date of the laboratory result of glycaemia <4.0 mmol/L.

### 2.4 Statistical analyses

Mean and standard deviations for continuous variables, and number and proportions for categorical variables were calculated, stratified by exposure groups. We used a multiple logistic regression model to estimate the adjusted odds ratios (OR) and 95% confidence interval (CI) of the primary composite end‐point for a mean glycaemia of 4.0–7.0 mmol/L and a mean glycaemia of >10.0 mmol/L versus a mean glycaemia of 7.1–10.0 mmol/L. In secondary analyses, we used a Cox proportional hazards model to estimate the adjusted hazard ratios (HR) and corresponding 95% CI of hypoglycaemia for a mean glycaemia of 4.0–7.0 mmol/L and a mean glycaemia of >10.0 mmol/L versus a mean glycaemia of 7.1–10.0 mmol/L. To determine the risk of adverse events associated with hypoglycaemia during hospitalization, we used a multiple logistic regression model to estimate the adjusted OR and 95% CI of the primary composite end‐point in association with hypoglycaemia during hospitalization. All models were adjusted for the following potential confounding: age, sex, serum creatinine level measured at time of admission, use of cholesterol‐lowering agents, antihypertensives, diuretics, antiplatelets, anticoagulants and glucocorticoids documented at the time of admission.

### 2.5 Sensitivity analyses

The primary analysis was performed to assess the association between having a mean glycaemia of 4.0–7.0 mmol/L or a mean glycaemia of >10.0 mmol/L versus a mean glycaemia of 7.1–10.0 mmol/L during the first four days of hospitalization and the risk of all‐cause mortality. The primary and secondary analyses were repeated using average glycaemia during the entire hospitalization rather than only the first 4 days of hospitalization, to assess the association with the risk of primary composite outcome and hypoglycaemia.

## 3 RESULTS

A total of 1368 patients were included in the study. The distributions for age, sex and serum creatinine were comparable among the groups (Table 1). Patients with a mean glycaemia of 4.0–7.0 mmol/L had lower prevalence of use of anti‐diabetic medications, cholesterol‐lowering medications, anti‐hypertensive medications, diuretics, antiplatelets and glucocorticoids during hospitalization, compared to patients in the other two exposure categories. The average length of hospitalization was 18 ± 27 days in the mean glycaemia 4.0–7.0 mmol/L group, 16 ± 19 days in the mean glycaemia 7.1–10.0 mmol/L group and 15 ± 23 days in the mean glycaemia >10.0 mmol/L group.

**TABLE 1:** Baseline characteristics of patients with mean glycaemia of 4.0–7.0, 7.1–10.0 and >10.0 mmol/L during the first 4 days of hospitalization

Characteristics	Glucose 4.0–7.0 mmol/L		Glucose 7.1–10.0 mmol/L		Glucose >10.0 mmol/L		Entire cohort	
	n or mean	% or SD	n or mean	% or SD	n or mean	% or SD	n or mean	% or SD
Number of patients, n (%)	270	19.7	565	41.3	533	39	1368	100
Glucose, mean (SD)	6.1	0.7	8.5	0.8	12.7	2.3	9.6	3
Length of stay, days (SD)	18	27	16	19	15	23	16	23
Age (years), mean (SD)	69.6	15.2	70.3	15.3	71.6	14	70.7	14.8
18–40, n (%)	12	20.7	32	55.2	14	24.1	58	4.2
41–50, n (%)	15	27.3	19	34.6	21	38.2	55	4.0
51–60, n (%)	47	23.7	78	39.4	73	36.9	198	14.5
61–70, n (%)	60	18.5	133	41.1	131	40.4	324	23.6
71–80, n (%)	67	19.3	145	41.7	136	39.1	348	25.4
81–90, n (%)	50	16.2	130	42.2	128	41.6	308	22.5
91+, n (%)	19	24.7	28	36.4	30	39	77	5.6
Male, n (%)	156	57.8	318	56.3	304	57	780	57.0
Serum creatinine (μmol/L), mean (SD)	148	161.4	170.2	169	162.4	163.4	162.6	165.3
Anti‐diabetic use, n (%)	141	52.2	450	79.7	494	92.7	1085	79.3
⍺ glucosidase inhibitors	1	0.4	2	0.4	3	0.6	6	0.4
DPP−4 inhibitors^a^	37	13.7	132	23.4	178	33.4	347	25.4
GLP−1 agonists^b^	2	0.7	2	0.4	5	0.9	9	0.7
Insulin	57	21.1	216	38.2	316	59.3	589	43.1
Meglitinides	6	2.2	13	2.3	10	1.9	29	2.1
Metformin	98	36.3	275	48.7	318	59.7	691	50.5
SGLT2 inhibitors^c^	1	0.4	23	4.1	9	1.7	33	2.4
Sulfonylureas	26	9.6	114	20.2	149	28.0	289	21.1
Thiazolidinediones	1	0.4	9	1.6	3	0.6	13	1.0
Anti‐coagulant use, n (%)	78	28.9	157	27.8	166	31.1	402	29.4
Anti‐hypertensive use, n (%)	187	69.3	456	80.7	415	77.9	1060	77.5
Antiplatelet use, n (%)	105	38.9	264	46.7	248	46.5	617	45.1
Diuretic use, n (%)	94	34.8	263	46.6	262	49.2	620	45.3
Glucocorticoid use, n (%)	47	17.4	132	23.4	137	25.7	316	23.1
Hypolipidemic use, n (%)	141	52.2	364	64.4	333	62.5	838	61.3

^a^ DPP‐4 inhibitors: Dipeptidyl peptidase‐4 inhibitors.

^b^ GLP‐1 agonists: Glucagon‐like peptide‐1 receptor agonists.

^c^ SGLT2 inhibitors: Sodium‐glucose cotransporter‐2 inhibitors.

A total of 407 patients (29.8%) experienced the adverse event composite end‐point (Table 2). The cumulative risk of the composite end‐point was 30.5% among those with a mean glycaemia of 7.1–10.0 mmol/L, 31.0% among those with a mean glycaemia of >10.0 mmol/L and 28.0% among those with a mean glycaemia of 4.0–7.0 mmol/L. Compared with a mean glycaemia of 7.1–10.0 mmol/L, a mean glycaemia of 4.0–7.0 mmol/L during the first 4 days of hospitalization was not associated with the occurrence of the primary composite end‐point (adjusted OR 0.88, 95% CI 0.63–1.23). Similarly, a mean glycaemia of >10.0 mmol/L during the 4 days of hospitalization was not associated with the occurrence of the primary composite end‐point (adjusted OR 0.98, 95% CI 0.75–1.28). The majority of adverse events recorded was all‐cause mortality in all three exposure groups (Table S1).

**TABLE 2:** Crude and adjusted odds ratios for the association between mean glycaemia in the first four days and the risk of adverse outcomes during hospitalization^a^

Mean glycaemic level (mmol/L)	Number of patients with composite primary outcome (%)	Number of patients at risk	Crude OR (95% CI)	Adjusted OR (95% CI)
4.0–7.0	74 (28.0)	264	0.89 (0.64, 1.23)	0.88 (0.63, 1.23)
7.1–10.0	170 (30.5)	557	Reference	Reference
> 10.0	163 (31.0)	525	1.03 (0.79, 1.33)	0.98 (0.75, 1.28)

Abbreviations: CI, confidence intervals; OR, odds ratio.

^a^ 22 observations were deleted due to missing values (6 from the mean glycaemia 4.0–7.0 mmol/L group, 8 from the mean glycaemia 7.1–10.0 mmol/L group and 8 from the mean glycaemia >10.0 mmol/L group). Analyses were adjusted for age, sex, creatinine level, use of cholesterol‐lowering agents, antihypertensives, antiplatelets, anticoagulants and glucocorticoids.

Compared with a mean glycaemia of 7.1–10.0 mmol/L, a mean glycaemia of >10.0 mmol/L during the first four days of hospitalization was associated with an increased risk of hypoglycaemia (adjusted HR 1.72, 95% CI 1.21–2.45) (Table 3). In contrast, a mean glycaemia of 4.0–7.0 mmol/L was not associated with an increased risk of hypoglycaemia (adjusted HR 1.29, 95% CI 0.84–1.98). Hypoglycaemia during hospitalization was associated with an increased risk of the primary composite outcome (adjusted OR 1.85, 95% CI 1.31–2.60) (Table 4).

**TABLE 3:** Crude and adjusted hazard ratios for the association between average glycaemia in the first 4 days and the risk of hypoglycaemia during hospitalization^a^

Mean glycaemic level (mmol/L)	Number of hypoglycaemia events (%)	Number of patients at risk	Crude HR (95% CI)	Adjusted HR (95% CI)
4.0–7.0	36 (13.6)	265	1.34 (0.88, 2.04)	1.29 (0.84, 1.98)
7.1–10.0	55 (9.9)	557	Reference	Reference
> 10.0	75 (14.3)	525	1.76 (1.24, 2.49)	1.72 (1.21, 2.45)

Abbreviations: CI, confidence intervals; HR, hazard ratio.

^a^ 21 observations were deleted due to missing values (5 from the mean glycaemia 4.0–7.0 mmol/L group, 8 from the mean glycaemia 7.1–10.0 mmol/L group and 8 from the mean glycaemia >10.0 mmol/L group). Analyses were adjusted for age, sex, creatinine level, use of cholesterol‐lowering agents, antihypertensives, antiplatelets, anticoagulants and glucocorticoids.

**TABLE 4:** Crude and adjusted odds ratios for the association between hypoglycaemia and the risk of adverse outcomes during hospitalization^a^

Occurrence of hypoglycaemia	Number of patients with composite primary outcome	Number of patients at risk	Crude OR	Adjusted OR
			(95% CI)	(95% CI)
No	338	1181	Reference	Reference
Yes	71	167	1.85 (1.32, 2.57)	1.85 (1.31, 2.60)

Abbreviations: CI, confidence intervals; OR, odds ratio.

^a^ 20 observations were deleted due to missing values (20 from the no occurrence of hypoglycaemia group and 0 from the occurrence of hypoglycaemia group). Analyses were adjusted for age, sex, creatinine level, use of cholesterol‐lowering agents, antihypertensives, antiplatelets, anticoagulants and glucocorticoids.

Sensitivity analysis assessing the risk of all‐cause mortality associated with a mean glycaemia of 4.0–7.0 mmol/L and >10.0 mmol/L compared to a mean glycaemia of 7.1–10.0 mmol/L resulted in consistent findings (mean glycaemia 4.0–7.0 mmol/L adjusted OR: 0.86; 95% CI: 0.61–1.20 and mean glycaemia >10.0 mmol/L adjusted OR: 0.99; 95% CI: 0.76–1.30) (Table S2). Additional sensitivity analyses were performed with mean glycaemia from the entire hospitalization. Of note, the mean glucose in the three groups using glycaemic data from the entire hospitalization was comparable to using glycaemic data from the first four days of hospitalization (Table S3). Similar to the primary analysis findings, there was no association between average glycaemia during the entire hospitalization and the risk of primary composite outcome (Table S4). There was no association between average glycaemia during the entire hospitalization and the risk of hypoglycaemia (Table S5).

## 4 DISCUSSION

In this study, there was no association between a mean glycaemia of 4.0–7.0 mmol/L or a mean glycaemia of >10.0 mmol/L and risk of adverse events among hospitalized patients in non‐critical care internal medicine units, compared to patients with a mean glycaemia of 7.1–10.0 mmol/L. The mean length of hospitalization was similar between patients in the three glycaemia categories. Having a mean glycaemia of >10.0 mmol/L during the first four days of hospitalization was associated with a 72% increased risk of hypoglycaemia. Hypoglycaemia during hospitalization was associated with a nearly twofold higher risk of adverse events.

Although there is growing clinical evidence indicating the need for treating hyperglycaemia among hospitalized patients with diabetes, the management of hyperglycaemia is challenging, and the optimal glycaemia target has not been well studied.^25^ Currently suggested targets are difficult to achieve given that it requires more effort and the risk of hypoglycaemia may increase when the glycaemia is targeted to a lower range within normal. Clinical studies have shown that tight glycaemic control may improve outcomes among patients with acute coronary syndrome via reducing oxidative stress and inflammation..^30^, ^31^, ^32^ However, two large randomized clinical trials, DIGAMI and NICE‐SUGAR, which involved cardiac and intensive care patients, respectively, have provided conflicting results on the risk of mortality associated with intensive glycaemic control during hospitalization.^33^, ^34^ Thus, the practice of using intensive insulin therapy to achieve tight glycaemic control among critically ill patients has not been justified by these studies given such practice may not improve mortality and can increase the risk of hypoglycaemia.^34^ Studies performed so far on non‐critically ill patients are fewer, and most studies have shown an increased risk of adverse outcomes among hospitalized patients with hyperglycaemia.^1^, ^4^, ^5^, ^6^, ^7^, ^8^, ^9^, ^10^, ^11^, ^26^ In our study, having a glycaemia of >10.0 mmol/L was not found to be associated with an increased risk of adverse outcomes. The meta‐analysis by Murad et al.^25^ demonstrated similar results; intensive glycaemic control, defined largely by fasting blood glucose level between 5.6 to 10 mmol/L, was not associated with reductions of mortality, myocardial infarction or stroke risks. However, this meta‐analysis found an association between intensive glycaemic control and reduced infection risk, predominantly in surgical patients.

There are a few possible explanations for the null finding in our study. First, our sample size may have been insufficient to allow for the detection of smaller but clinically important differences in the risk of adverse events among different glycaemic control groups. Second, the internal medicine units in this study are teaching units staffed by a large team of attending physicians, resident physicians and medical students. As such, there are usually actions taken to address hyperglycaemia in a timely fashion. Patients with abnormal test results such as hyperglycaemia may have received more medical attention.

The findings from our secondary outcome suggest that having a glycaemia of >10.0 mmol/L during the 4 days of hospitalization was associated with a higher risk of hypoglycaemia, whereas having a glycaemia of 4.0–7.0 mmol/L was not associated with an increased risk of hypoglycaemia. In the literature, having lower glycaemia within the recommended target range is generally associated with a higher risk of hypoglycaemia, with most evidence derived from critically ill or post‐myocardial infarction patients.^35^, ^36^, ^37^ In our study, the percentage of patients with mean glycaemia 4.0–7.0 mmol/L who experienced hypoglycaemia is similar to that of patients with the mean glycaemia >10.0 mmol/L; our sample size may have been insufficient to allow detection of significant hypoglycaemia risk in those with the mean glycaemia of 4.0–7.0 mmol/L. The increased hypoglycaemia risk in patients with a mean glycaemia of >10.0 mmol/L during the first four days of hospitalization may be due to usage of insulin and particularly insulin sliding scale, to treat hyperglycaemia and that these patients are more susceptible to hypoglycaemia with insulin treatment. Patients with a mean glycaemia of >10.0 mmol/L during the entire hospitalization were not found to be at increased risk of hypoglycaemia, suggesting that patients with hyperglycaemia throughout hospitalization do not have an increased risk of hypoglycaemia. Intensive insulin therapy has been shown to be associated with an increased risk of hypoglycaemia in the critical care setting.^38^ Furthermore, insulin sliding scale has been shown in some studies to increase the risk of both hyperglycaemia and hypoglycaemia.^39^ Further studies are needed to understand the relationship between glycaemic control during hospitalization and hypoglycaemia risk.

Hypoglycaemia during hospitalization was associated with a nearly twofold increase in the risk of adverse outcomes. Hypoglycaemia during hospitalization has been associated with an increased risk of all‐cause mortality (HR: 2.55; 95% CI: 2.25–2.88) in a retrospective study of patients aged ≥66 years over a 4 year follow‐up period.^40^ Hypoglycaemia has been shown to be associated with an increased risk of vascular outcomes and all‐cause mortality in the outpatient setting.^41^, ^42^ Although it is possible that hypoglycaemia may contribute to increased risks of vascular complications and all‐cause mortality, hypoglycaemia may also act as a marker of increased comorbidity and thus predicts a higher risk of all‐cause mortality. Nevertheless, the findings from our study suggest that hypoglycaemia should be avoided during hospitalization. Further studies are warranted to determine whether there is a causal relationship between hypoglycaemia and an increased risk of adverse outcomes during hospitalization.

Interestingly, we found an association between having a mean glycaemia of >10.0 mmol/L and increased hypoglycaemia risk, and an association between hypoglycaemia and risk of adverse events, while there was no association between having mean glycaemia of >10.0 mmol/L and risk of adverse events. Possible explanations for this include that there are factors other than the proposed use of insulin and insulin sliding scale contributing to hypoglycaemia, for example terminal frailty, unreliable oral intake or severe underlying illnesses that is associated with hypoglycaemia and an increased risk of death. Thus, iatrogenic hypoglycaemia is unlikely to be associated with increased adverse events compared to spontaneous hypoglycaemia.^42^ Further studies are needed to assess glycaemic control and risk of hypoglycaemia during hospitalization.

This study has some strengths. First, to our knowledge, this is the first study to assess various glycaemia cut‐offs within the recommended glycaemia target to further discern optimal glycaemia management during non‐critical care hospitalization. Second, we were able to adjust for a number of confounders, including usage of a few medication classes which are reflective of underlying patient comorbidities. Third, in order to minimize protopathic bias, glycaemic control was calculated based on glycaemia during the first four days of hospitalization only.

This study also has limitations. First, patients with various types of diabetes were included in the study. The current in‐hospital glycaemia targets in guidelines do not distinguish types of diabetes. However, it is reasonable to suspect that adverse effects of hyperglycaemia may differ based on the underlying mechanism causing hyperglycaemia. Second, as the vast majority of adverse events recorded were deaths, the number of events was insufficient to examine the risks of the individual components of our composite end‐point (Table S1). Third, we were unable to obtain the date of adverse events that occurred during hospitalization. Therefore, in our primary analysis, some adverse events may have happened during the first four days of hospitalization, leading to potential risk of reverse causality. However, the risk of reverse causality unlikely had significant contribution to the results, as most adverse events recorded were mortalities (Table S1). Fourth, measurements for severity of diabetes and insulin dosing used during hospitalization were not available in our databases, and thus, the analyses did not adjust for diabetes severity or duration. Similarly, we do not have information on the nutritional status of the patients during hospitalization, which may affect glycaemic control. Fifth, we were unable to adjust for the severity of patients’ presenting illness due to the heterogeneity of admission diagnoses. Sixth, the averaged glucose values do not account for situations where glucose may have been repeatedly checked over a short period of time, for example when treating hypoglycaemia, which may have skewed the mean glucose values. However, only 20 patients had hypoglycaemia during the first four days of hospitalization. Seventh, the study focussed on glycaemic control and adverse events during hospitalization. As such, some complications such as mortality, cardiovascular events, infection may take time to develop and may happen after a hospitalization. Unfortunately, our study was unable to assess outcomes that occurred after hospital discharge. Finally, as this is an observational study, there may be residual confounding.

## 5 CONCLUSIONS

For non‐critically ill patients hospitalized on internal medicine units, neither having a mean glycaemia of 4.0–7.0 mmol/L nor a mean glycaemia of >10 mmol/L was associated with increased risks of adverse events. The result for having a mean glycaemia of >10 mmol/L was unexpected and may be due to increased medical attention given to these patients and timely intervention given to lower glycaemia. Mean glycaemia of >10 mmol/L was associated with a higher risk of hypoglycaemia, likely attributable to aggressive glucose lowering measures, arguing for more attention on hyperglycaemia management in hospital. Hypoglycaemia during hospitalization is associated with a nearly twofold increase in the risk of adverse events, which may be associated with increased disease severity and emphasizes the need to avoid hypoglycaemia during hospitalization. This study highlights the need for further studies on optimal glycaemic target in the non‐critically ill patient population.

## CONFLICT OF INTEREST

All authors declare no conflict of interests relevant to this study.

## AUTHOR CONTRIBUTIONS

Lan Deng wrote the manuscript. Wusiman Aibibula performed data analysis. Zahra Talat and Shaun Eintracht performed the data collection. All authors contributed to study design, interpretation of data, and reviewed and approved the final manuscript. Oriana Hoi Yun Yu is the guarantor of this work, had full access to the data and takes responsibility for the integrity of the data and data analyses.

## Supporting information

Table S1‐S5

## Data Availability

The data used that supports the findings of this study are available from the corresponding author upon reasonable request and after approval is obtained to release data from the Research Ethics Committee at the Jewish General Hospital, Montréal, Canada.
