# Supplementary material for: The association between glycaemic control during hospitalization and risk of adverse events: A retrospective cohort study
Source: Endocrinol Diabetes Metab. 2021 May 27;4(3):e00268. doi: 10.1002/edm2.268 (PMC8279636; doi:10.1002/edm2.268)
Supplement: Supplementary file 1 — Table S1‐S5 [file EDM2-4-e00268-s001.docx]

| **Supplemental Table 1. Breakdown of adverse events with glycemic control^a^** | | | |
| --- | --- | --- | --- |
|  | **Mean glycemia (mmol/L)** | | |
|  | **4.0 – 7.0** | **7.1 – 10.0** | **> 10.0** |
| **Number of patients with composite primary outcome (%)** | 74 (28.0) | 170 (30.5) | 163 (31.0) |
| **Number of patients at risk** | 264 | 557 | 525 |
| **Adjusted OR (95% CI)** | 0.88 (0.63, 1.23) | Reference | 0.98 (0.75, 1.28) |
| **Breakdown of adverse events** |  | | |
| **Acute kidney injury** | 3 | 6 | 2 |
| **Cellulitis** | 0 | 0 | 1 |
| **Clostridium difficile and other infectious colitis** | 0 | 2 | 0 |
| **Deep vein thrombosis** | 0 | 0 | 0 |
| **Ischemic stroke** | 0 | 1 | 0 |
| **Myocardial infarction** | 1 | 6 | 5 |
| **Pneumonia** | 0 | 1 | 0 |
| **Pulmonary embolism** | 0 | 0 | 0 |
| **Sepsis** | 0 | 0 | 0 |
| **Septic shock** | 0 | 0 | 0 |
| **Transient ischemic attack** | 0 | 0 | 0 |
| **Unstable angina** | 0 | 0 | 0 |
| **Urinary tract infection** | 0 | 0 | 1 |
| **Wound ulcer** | 0 | 0 | 0 |
| **Death** | 70 | 162 | 157 |
| ^a^22 observations were deleted due to missing values (6 from the mean glycemia 4.0-7.0 mmol/L group, 8 from the mean glycemia 7.1-10.0 mmol/L group, and 8 from the mean glycemia > 10 mmol/L group). | | | |

| **Supplemental Table 2. Crude and adjusted odds ratios for the association between mean glycemia during the first four days of hospitalization and the risk of all-cause mortality^a^** | | | | |
| --- | --- | --- | --- | --- |
| **Mean glycemic level (mmol/L)** | **Number of patients with mortality (%)** | **Number of patients at risk** | **Crude OR**  **(95% CI)** | **Adjusted OR**  **(95% CI)** |
| **4.0-7.0** | 70 (26.4) | 265 | 0.88 (0.63, 1.22) | 0.86 (0.61, 1.20) |
| **7.1-10.0** | 162 (29.1) | 557 | Reference | Reference |
| **> 10** | 157 (29.9) | 525 | 1.04 (0.80, 1.35) | 0.99 (0.76, 1.30) |
| Abbreviations: CI, confidence intervals; OR, odds ratio | | | | |
| ^a^21 observations were deleted due to missing values (5 from the mean glycemia 4.0-7.0 mmol/L group, 8 from the mean glycemia 7.1-10.0 mmol/L group, and 8 from the mean glycemia > 10 mmol/L group). Analyses were adjusted for age, sex, creatinine level, use of cholesterol-lowering agents, antihypertensives, antiplatelets, anticoagulants, and glucocorticoids. | | | | |

| **Supplemental Table 3. Baseline characteristics in patients with mean glycemia of 4.0-7.0, 7.1-10, and > 10 mmol/L during the entire hospitalization** | | | | | | | | |
| --- | --- | --- | --- | --- | --- | --- | --- | --- |
| **Characteristics** | **Glucose 4.0-7.0 mmol/L** | | **Glucose 7.1-10.0 mmol/L** | | **Glucose >10 mmol/L** | | **Entire cohort** | |
|  | **n or mean** | **% or SD** | **n or mean** | **% or SD** | **n or mean** | **% or SD** | **n or mean** | **% or SD** |
| **Number of patients, n (%)** | 253 | 18.6 | 615 | 44.9 | 500 | 36.5 | 1368 | 100 |
| **Glucose, mean (SD)** | 6.2 | 0.6 | 8.5 | 0.9 | 12.2 | 1.9 | 9.4 | 2.6 |
| **Age (years), mean (SD)** | 69.5 | 16.0 | 70.5 | 14.8 | 71.4 | 14.1 | 70.7 | 14.8 |
| **18-40, n (%)** | 15 | 25.9 | 30 | 51.7 | 13 | 22.4 | 58 | 4.2 |
| **41-50, n (%)** | 14 | 25.4 | 20 | 36.4 | 21 | 38.2 | 55 | 4.0 |
| **51-60, n (%)** | 37 | 18.7 | 93 | 47.0 | 68 | 34.3 | 198 | 14.4 |
| **61-70, n (%)** | 56 | 17.3 | 140 | 43.2 | 128 | 39.5 | 324 | 23.7 |
| **71-80, n (%)** | 65 | 18.7 | 162 | 46.6 | 121 | 34.8 | 348 | 25.4 |
| **81-90, n (%)** | 50 | 16.2 | 139 | 45.1 | 119 | 38.6 | 308 | 22.5 |
| **91+, n (%)** | 16 | 20.8 | 31 | 40.3 | 30 | 39.0 | 77 | 5.6 |
| **Male, n (%)** | 141 | 55.7 | 358 | 58.2 | 279 | 55.8 | 778 | 56.9 |
| **Serum creatinine, mean (SD)** | 137.4 | 155.5 | 167.5 | 162.5 | 169.7 | 172.8 | 162.8 | 165.4 |
| **Anti-diabetic use, n (%)** | 116 | 45.9 | 498 | 81.0 | 471 | 94.2 | 1085 | 79.3 |
| **⍺ glucosidase inhibitors** | 0 | 0.0 | 3 | 0.5 | 3 | 0.6 | 6 | 0.4 |
| **DPP-4 inhibitors^a^** | 28 | 11.1 | 145 | 23.6 | 174 | 34.8 | 347 | 25.4 |
| **GLP-1 agonists^b^** | 1 | 0.4 | 4 | 0.7 | 4 | 0.8 | 9 | 0.7 |
| **Insulin** | 41 | 16.2 | 234 | 38.1 | 314 | 62.8 | 589 | 43.1 |
| **Meglitinides** | 4 | 1.6 | 16 | 2.6 | 9 | 1.8 | 29 | 2.1 |
| **Metformin** | 81 | 32.0 | 319 | 51.9 | 291 | 58.2 | 691 | 50.4 |
| **SGLT2 inhibitors^c^** | 1 | 0.4 | 20 | 3.3 | 12 | 2.4 | 33 | 2.4 |
| **Sulfonylureas** | 17 | 6.7 | 130 | 21.1 | 142 | 28.4 | 289 | 21.1 |
| **Thiazolidinediones** | 1 | 0.4 | 8 | 1.3 | 4 | 0.8 | 13 | 1.0 |
| **Anti-coagulant use, n (%)** | 77 | 30.4 | 170 | 27.6 | 154 | 30.8 | 401 | 29.3 |
| **Anti-hypertensive use, n (%)** | 173 | 68.4 | 488 | 79.4 | 397 | 79.4 | 1058 | 77.3 |
| **Anti-platelet use, n (%)** | 98 | 38.7 | 283 | 46.0 | 236 | 47.2 | 617 | 45.1 |
| **Diuretic use, n (%)** | 87 | 34.4 | 287 | 46.7 | 245 | 49.0 | 619 | 45.3 |
| **Glucocorticoid use, n (%)** | 49 | 19.4 | 135 | 22.0 | 132 | 26.4 | 316 | 23.1 |
| **Hypolipidemic use, n (%)** | 123 | 48.6 | 391 | 63.6 | 324 | 64.8 | 838 | 61.3 |
| ^aa^DPP-4 inhibitors: Dipeptidyl peptidase-4 inhibitors ^b^GLP-1 agonists: Glucagon-like peptide-1 receptor agonists ^c^SGLT2 inhibitors: Sodium-glucose cotransporter-2 inhibitors | | | | | | | | |

| **Supplemental Table 4. Crude and adjusted odds ratios for the association between mean glycemia during the entire hospitalization and the risk of adverse outcomes^a^** | | | | |
| --- | --- | --- | --- | --- |
| **Mean glycemic level (mmol/L)** | **Number of patients with composite primary outcome (%)** | **Number of patients at risk** | **Crude OR**  **(95% CI)** | **Adjusted OR**  **(95% CI)** |
| **4.0-7.0** | 64 (25.9) | 247 | 0.82 (0.59, 1.15) | 0.81 (0.57, 1.14) |
| **7.1-10.0** | 181 (29.8) | 607 | Reference | Reference |
| **> 10.0** | 162 (32.9) | 492 | 1.16 (0.89, 1.49) | 1.12 (0.86, 1.46) |
| Abbreviations: CI, confidence intervals; OR, odds ratio | | | | |
| ^a^22 observations were deleted due to missing values (6 from the mean glycemia 4.0-7.0 mmol/L group, 8 from the mean glycemia 7.1-10.0 mmol/L group, and 8 from the mean glycemia > 10.0 mmol/L group). Analyses were adjusted for age, sex, creatinine level, use of cholesterol-lowering agents, antihypertensives, antiplatelets, anticoagulants, and glucocorticoids. | | | | |

| **Supplemental Table 5. Crude and adjusted hazard ratios for the association between average glycemia during the entire hospitalization and the risk of hypoglycemia^a^** | | | | |
| --- | --- | --- | --- | --- |
| **Mean glycemic level (mmol/L)** | **Number of hypoglycemia events (%)** | **Number of patients at risk** | **Crude HR**  **(95% CI)** | **Adjusted HR**  **(95% CI)** |
| **4.0-7.0** | 25 (10.1) | 248 | 0.80 (0.51, 1.26) | 0.75 (0.48, 1.19) |
| **7.1-10.0** | 76 (12.5) | 607 | Reference | Reference |
| **> 10.0** | 65 (13.2) | 492 | 1.28 (0.92, 1.79) | 1.30 (0.93, 1.81) |
| Abbreviations: CI, confidence intervals; HR, hazard ratio | | | | |
| ^a^21 observations were deleted due to missing values (5 from the mean glycemia 4.0-7.0 mmol/L group, 8 from the mean glycemia 7.1-10.0 mmol/L group, and 8 from the mean glycemia > 10.0 mmol/L group). Analyses were adjusted for age, sex, creatinine level, use of cholesterol-lowering agents, antihypertensives, antiplatelets, anticoagulants, and glucocorticoids. | | | | |
